# Perspectives of syringe services program operators in Michigan on their relationship with substance use treatment: a qualitative study

**DOI:** 10.1186/s12954-025-01172-5

**Published:** 2025-03-01

**Authors:** Molly C. Reid, Samantha J. Harris, Suzanne M. Grieb, Sabrina Gattine, Zekiye Lukco, Brandon Hool, Mary Aguirre, Fernanda Alonso Aranda, Catherine Tomko, Sara Whaley, Brendan Saloner, Sean T. Allen

**Affiliations:** 1 Department of Mental Health, Johns Hopkins University Bloomberg School of Public Health, 624 N Broadway, Baltimore, MD 21205 USA; 2 Department of Health Policy and Management, Johns Hopkins University Bloomberg School of Public Health, Baltimore, MD 21205 USA; 3 Center for Child and Community Health Research, Johns Hopkins University School of Medicine, Baltimore, MD 21224 USA; 4 Department of Health, Behavior and Society, Johns Hopkins University Bloomberg School of Public Health, Baltimore, MD 21205 USA; 5 Michigan Department of Health and Human Services, Lansing, MI 48909 USA; 6 Vital Strategies, New York, NY 10005 USA

**Keywords:** Harm reduction, Substance use treatment, Integrated services

## Abstract

**Background:**

Substance use treatment (SUT) and harm reduction are often perceived as having distinct goals despite people who use drugs routinely having needs that encompass both services. The co-occurring SUT and harm reduction needs of people who use drugs warrant collaboration between service providers. However, little work has explored such collaborations, or lack thereof. This research explores how SUT providers responded to expanded harm reduction programming from the perspectives of syringe services program (SSP) operators in Michigan.

**Methods:**

We conducted in-depth, semi-structured interviews with a geographically diverse sample of SSP operators (*n* = 19) in Michigan during October and November 2021. The interview guide broadly explored the contributing factors to SSP implementation, including SSP relationships with SUT providers. Analyses of transcribed interviews were conducted using an iterative, thematic constant comparison process informed by grounded theory.

**Results:**

Participants described a range of responses to SSP implementation from SUT providers. Many SSP operators identified significant barriers to effective collaboration with SUT providers due to lack of awareness about harm reduction approaches and stigmatization of drug use. For example, SUT providers were often reluctant to accept free harm reduction supplies (e.g., sterile syringes, naloxone) to offer their clients. Participants also reported difficulties connecting their clients to evidence-based SUT providers. Incremental relationship building and education about the role of harm reduction in recovery were required for effective collaboration between SUT providers and SSPs.

**Conclusions:**

Tensions between SUT and harm reduction providers may create challenges that impede recovery among people who use drugs. Ensuring SUT and harm reduction providers understand the unique and complementary roles of each approach is necessary to advance the health of people who use drugs.

## Introduction

The United States (US) is facing an epidemic of overdose mortality related to opioid use, with overdose deaths reaching an all-time high in 2021 [1]. Injection drug use (IDU) is a significant risk factor for overdose and bloodborne infection, including HIV and HCV [2]. Harm reduction initiatives, such as syringe services programs (SSPs), are instrumental in combatting the overdose crisis and mitigating bloodborne infectious disease transmission associated with high-risk injection practices [3]. SSPs provide sterile syringes to people who use drugs (PWUD) in addition to other services such as syringe disposal, injection equipment access, and connection to medical care and other supports [4]. Since the 1980s, research has documented the benefits of SSPs in reducing infectious disease transmission [5, 6], reducing syringe sharing [7], and increasing linkages to substance use treatment (SUT) [8]. Despite the robust evidence in support of harm reduction interventions, their adoption is not widespread in the US. Harm reduction interventions can face considerable opposition due to the stigmatization of drug use [5].

The disapprobation of harm reduction in the US persists even in settings designated to provide care for PWUD. While SUT and harm reduction are both integral components of a comprehensive public health approach to mitigating substance use related harms, there can be tension between SUT providers and harm reductionists [9–11]. For example, nearly a quarter of SUT providers reported that providing sterile syringes to PWUD was somewhat or completely unacceptable [9]. Non-acceptance of syringe services and other harm reduction interventions among SUT providers has also been associated with stigmatizing beliefs about drug use [9, 12]. In part, these beliefs may stem from the legacy of “abstinence-only” philosophies in substance use programming and prevention initiatives [9, 11]. Despite the emphasis on abstinence in many SUT programs, comparative studies show that treatment models which incorporate medications for opioid use disorder (MOUD), particularly with psychosocial supports, are a more effective, evidence-based approach for substance use [13, 14].

Between 2017 and 2022, Michigan scaled up the number of SSPs operating throughout the state from four to 35 programs [15]. This rapid expansion of harm reduction services was funded as part of the Michigan Department of Health and Human Services’ effort to address statewide increases in overdose mortality and IDU-related infectious disease transmission. In 2020, the state reported 2,759 overdose deaths, an 18-fold increase from 1999 [16–18]. In 2021, Michigan identified 633 new HIV infections [18].

While Michigan does not prohibit the distribution of drug paraphernalia such as syringes, SSPs are not explicitly authorized under state law, creating a legal grey area within which harm reduction services must operate [15]. Programs that distribute syringes are not legally restricted from doing so, but the lack of explicit legislative language authorizing SSPs may add a layer of uncertainty and liability to organizations considering this service [19]. Though the state has made substantial investments in improving SSP availability, its SUT options are also lacking. Michigan has the twelfth lowest percentage of treatment facilities offering MOUD in the country, underscoring an overreliance on non-evidence-based treatment models [20].

The relationship between harm reduction and SUT is poorly understood and warrants further exploration to understand how to best meet the service needs of PWUD [21]. This analysis examines the relationship between new SUT programs and harm reduction services in Michigan from the perspectives of SSP operators. We interviewed SSP operators about their experiences collaborating with SUT providers upon launching their SSP to better understand barriers and facilitators to SSPs and treatment programs working together to address the overdose crisis.

## Methods

We conducted in-depth, semi-structured interviews from October and November 2021 with staff who operated SSPs throughout the state of Michigan. The Michigan Department of Health and Human Services provided the [redacted for peer review] team with a list of potential SSP contacts. We also conducted online searches to identify potential SSP operators using publicly available SSP websites and news reports. Additionally, early participants recommended further potential SSP operators to contact for the study. Eligibility criteria included being at least 18 years of age and having played a role in SSP implementation in Michigan.

We contacted potential participants via e-mail or phone number, gave them basic information about the study, and asked if they would be interested in participating. Interested individuals were scheduled for a Zoom or telephone interview. Participants were able to choose where they completed the interview to ensure privacy. STA and SMG conducted the interviews, both of whom have formal education in conducting qualitative research, relevant interviewing experience, and familiarity with programs for PWUD. Interviews lasted approximately 45 min and were audio-recorded with participants’ permission. All participants were offered a $25 gift card as an incentive. Participants provided oral consent before interviews began. Interviews were transcribed verbatim. We began the analysis while the interviews were occurring, and stopped recruitment when content saturation was achieved. In other words, we stopped recruitment when we stopped learning new relevant information from interviews [20]. The Institutional Review Board at the Johns Hopkins Bloomberg School of Public Health determined this project did not constitute human subjects research as it focused on program development/evaluation.

The interview guide explored the experiences of SSP operators working with SUT providers during the scale-up of SSPs in Michigan. Our guide was informed by the Consolidated Framework for Implementation Research (CFIR) [21], as well as existing literature on SSP implementation [22–25]. Within the CFIR, we drew on constructs related to “partnerships and connections” of the outer setting domain, and the “relational connections”, “culture”, and “communications” constructs of the inner setting domain. After developing a preliminary interview guide, our collaborators at the Michigan Department of Health and Human Services worked with our team to tailor the guide to optimize its relevance for the local context and implementation setting. We subsequently conducted mock interviews with our study team to further refine the interview guide.

Data analysis of the text was conducted using an iterative, thematic constant comparison process informed by constructivist grounded theory [26, 27]. Two team members led the coding framework (SJH, SMG). SJH, MCR, and STA reviewed and discussed findings relating to harm reduction organization and substance use treatment program relations. SJH has a background in mixed-methods public health research, with expertise in the organization and delivery of substance use disorder and harm reduction services. SMG has expertise in community-engaged qualitative research on social determinants of health. MCR has a background in mixed methods research and substance use epidemiology. STA has expertise in mixed-methods research, particularly around harm reduction, drug policy, and HIV prevention. He also has more than a decade of experience conducting research on SSP implementation processes.

The analysis team brought extensive prior experience in the areas of harm reduction and substance use, and we employed several strategies to ensure the rigor of methodology and encourage reflexivity. Interviews were led by multiple interviewers, and the team met frequently to discuss emerging findings and find consensus across the collective team’s interpretations of the data [28, 29]. These regular meetings involved reflexive discussions, allowing us to limit potential biases and ensure results were based on the data collected in the current study rather than the team’s individual beliefs or experiences [30]. In addition, the analysis team discussed their findings with state partners to ensure analyses were accurately capturing the local context [31].

In order to develop the initial coding framework, two team members (SJH, SMG) independently conducted open coding on four transcripts and the results were merged and compared to create the initial codebook. The codebook went through multiple versions as more transcripts were coded and discussed by three qualitative coders (FAA, CT, GU). The interviews were independently coded using Atlas.ti software until each transcript had been coded twice. SJH reviewed the coded transcripts and resolved any discrepancies, ensuring intercoder agreement. To finalize the thematic structure, codes were compared both within and between interviews. Exemplary quotes have been deidentified to preserve participant anonymity, and results for key themes that emerged are reported.

## Results

Nineteen in-depth interviews with persons involved with SSP implementation in Michigan were completed. Among these participants, the median age was 38 years old. Most (73.7%; *n* = 14) identified as women. The majority identified as non-Hispanic and White. Participants held a diverse range of professional roles, including executive director, nurse, health officer, health educator, program operator, coordinator, manager, and program founder. We interviewed participants from SSPs run out of health departments and community based SSPs. Most participants referred to “treatment” and “recovery” providers as synonymous; we use “treatment providers” or “treatment community” to refer to those engaged with providing MOUD and/or counseling services to promote cessation of drug use.

### SSP staff were met with a mix of initial reactions by the treatment community

Participants described a range of reactions from the SUT community when they initially launched SSPs. For example, several participants emphasized that the SUT community was supportive of harm reduction programs, including SSPs:

They [the treatment community] obviously see that they don’t want people to have the endocarditis, hepatitis, HIV, things like that that are ongoing, so they’re pretty okay with it. I don’t think anybody that I’ve talked to has left being like “Oh, what a terrible idea to get people to start using [sterile] syringes and things.” I think that they’ve all seen the benefits

Support and a general sense of receptiveness toward harm reduction was particularly prevalent in smaller communities with fewer recovery resources. A participant from a rural area of Michigan explained this sentiment by stating:

We don’t have much of a recovery community here, honestly. […] But […] they’re willing to help with whatever. They’re very supportive and they love having the conversations

In contrast, many other SSP operators described the SUT community as initially reluctant to engage with SSP staff. In some cases, the SUT community refused to engage with the harm reduction providers.

They kind of shut down the conversations. […] They did not seem interested in shifting away from this recovery-focused narrative, and I remember even having a conversation in which I said “What if we ran one intervention with, say, ten of your clients that get the traditional and then we did ten that were more harm reduction-focused and we looked at what their outcomes were once the project ended.” They didn’t even want to engage in that […] they just weren’t even interested in those conversations…

### The treatment community was reluctant to offer harm reduction services, which SSP staff members attributed to a lack of treatment community understanding of the principles of harm reduction

Participants described scenarios in which their offers to integrate or collocate harm reduction programming at SUT facilities was not well-received. For example, several participants explained that they had offered to send harm reduction informational materials (such as flyers) to treatment providers, but were met with opposition.

I did talk to the [redacted] people to see if they would hand out my flyer that I made up when they discharge people from their programs, but they wouldn’t because they preach abstinence, not necessarily harm reduction

Other participants described situations in which treatment providers were against allowing SSP operators to provide free harm reduction supplies (e.g., naloxone) on their premises, or confiscating naloxone that patients bring to treatment with them.

A large part of our program is, of course, naloxone and Narcan distribution, which is what I call ‘safe harm reduction’ or ‘harm reduction light’. [But], they’re even taking folks’ [naloxone] kits [away from them] when they go inpatient. […] And because they’ve done that, they’ve actually had several overdoses within their facilities

SSP operators explained that offering free, sterile syringes at treatment facilities was met with significant opposition, including among programs that supported harm reduction conceptually.

We started partnering with some of the methadone clinics […] and for a while, some of them were okay with syringe distribution on the property and we had this really great kind of connection directly with the methadone clinic. […] But unfortunately,… doctors made a big stink about it and kind of swayed the opinion of the leaders of the clinics to then say, “You can no longer pass out syringes on the property,” to other addiction agencies who just said outright “No, you can’t do that. We support harm reduction, but we don’t support that harm reduction

When describing the treatment community’s reluctance to engage with SSPs, many interviewees highlighted the perceived dichotomy between treatment and harm reduction. They felt that this resulted from a lack of awareness of or belief in the effectiveness of harm reduction.

So, that’s how I kind of got into syringe service work or harm reduction work because the clinical addiction community is not harm reduction-oriented. Oftentimes it is engaged in practices that I’ve identified as harmful to the community, like removing people from treatment for continued drug use

This philosophical dichotomy was especially marked in the abstinence-oriented treatment communities:

Saying “the recovery community” is pretty broad, because there’s a lot of different factions within that, and there’s a lot of different beliefs within that as well, […] and I honestly think that the recovery community and treatment agencies is where a lot of our [harm reduction] work really needs to be done to continue moving things forward and have people who use drugs treated better in our communities… I think that a lot of harm has been done from the abstinence-only-based treatment and 12-steppers and such

In some cases, SSP staff described that treatment providers endorsed stigmatized views of harm reduction, including the perception that harm reduction enables drug use.

Because the attitude, and the attitude that still persists in recovery today is one that, “Well, you’re just enabling people, and you’re really not helping them.

### SSP staff were frustrated by limited and/or substandard treatment options to which to refer SSP clients interested in treatment

Overall, SSP operators described significant frustration with the scarcity of evidence-based SUT providers to which they could r nificant access barriers in some areas, as well as a lack of diversity in treatment modalities.

- *I think the biggest thing in rural communities and I will say this until the day that I die*, *is there are no treatment centers around here and if you want to go to treatment*, *you had better have Medicaid. If you can even find a bed because you cannot– There is not even community mental health here for people who have private insurance.*
- *We really wish that there were more options here just for treatment services across the board*, *just different providers*, *MAT [medication- assisted treatment]. We’re really*, *really lacking in options for MAT resources here*.

Several of the SSP staff also expressed concern with the “substandard” practices and evidence-base of the treatment center options in their areas.

*It can be difficult when you have a client who clearly needs help in some way but you don’t really have partners who you’re 100% sure are gonna treat this client the right way. An example is we had a client overdose and die a week and a half ago or so. [T]hey got kicked out of the methadone clinic*, *stopped having access to methadone and then started overdosing*, *and we helped them get into a treatment program*, *a detox and hopefully after that longer-term care*, *but the detox program pretty much dropped him.*

### In some instances, SSP staff were able to incrementally build relationships with the treatment community through provision of medical services, education, and developing shared understanding of substance use

Many interviewees identified strategies that helped overcome challenges to the integration of their harm reduction approaches within treatment communities. SSP staff emphasized the importance of incremental partnership building, including by offering less-controversial medical services to treatment programs, such as STI testing and vaccination.

I want to build partnerships. I want to build bridges and even if it’s just testing or even if I just come out there and do rapid HIV testing, it’s a great relationship because the addiction community is struggling with hepatitis C here and it’s a huge issue here. I’ll just keep talking to them and we’ll provide what they allow us to provide and hopefully in time, when they’re ready for syringe distribution on their property, they know that they can reach out to me and I’ll be there the very next day

While the treatment community was sometimes reluctant to host harm reduction providers or materials in their facilities, they were often more open to sending treatment staff to SSPs and offer resources and information to SSP clients. Some SSP staff felt that having treatment providers come on-site to SSPs also created an opening to start one-on-one conversations about harm reduction.

We’ve had some recovery coaches come out and meet with people and talk with people, and I think we’re still collaborating with more people and trying to get more people to come out with us […] It’s a matter of getting the recovery community to be able to come out with us, to actually sit there and talk to people. And I can talk to people about my own story and my own process, and that does help too. To actually have some one-on-one time and being able to relate to people has helped a lot

SSP staff also discussed the importance of connecting with treatment providers through problem recognition. When they focused on the public health issues related to substance use with treatment providers, they found a common ground from which the different approaches could be viewed as complementary.

- *I think with the overdose rates increasing*, *people are really coming around […] I think that’s a really big selling point. Dead people don’t recover*, *so if you want any chance of these people accessing recovery*, *they have to stay alive.”*
- *It would be beneficial if they kind of took a step back and saw the larger picture of like we all want to help the community and what I offer– this is what I tell everybody– what I offer is one part of a larger system. A syringe service program isn’t going to solve the opioid overdose epidemic in this country. It can’t. It’s too big of an epidemic to be solved through one avenue.*

## Discussion

Our qualitative study demonstrates the complex and evolving dynamics between two communities that often embrace different philosophical approaches to addressing substance use in Michigan. Many SSP operators described difficulty collaborating with SUT providers, including providers refusing to accept harm reduction supplies such as syringes and naloxone. Challenges with creating partnerships with SUT providers were attributed to a lack of awareness about harm reduction approaches and stigmatization of drug use among treatment providers. Through incremental relationship building and education about the role of harm reduction in recovery, in some instances, SSP operators were able to establish working relationships with treatment programs. Our study is one of very few to examine the relationship between harm reduction and treatment communities from the perspective of SSP staff, and highlights the importance of educating treatment providers about evidence-based approaches to substance use (e.g., offering harm reduction supplies, naloxone, and MOUD).

The initial pushback experienced by many of the SSP staff we spoke to is somewhat expected given the persistent disapproval of SSPs among treatment providers in the literature [9, 10]. SSP staff in Michigan may have been encountering “intervention stigma” of harm reduction, or stigmatizing beliefs about harm reduction interventions and both the providers and clients who are engaged in them [32]. Intervention stigma has been associated with staff reluctance to provide syringe services [33], perceived judgment of medical providers who provide take home naloxone [34], as well as negative and discriminatory experiences for PWUD seeking syringe services [35]. Low acceptance of harm reduction interventions among SUT providers is strongly correlated with endorsement of stigmatizing attitudes and beliefs about drug use in general [9, 12]. Addressing stigma of drug use and harm reduction interventions is imperative to reduce the impacts of the addiction and overdose crisis across the country.

The SSP staff we interviewed discussed the importance of educating the SUT community about harm reduction and how doing so advanced relationships that better served PWUD. They primarily referenced educating treatment providers through informal one-on-one conversations undertaken by themselves and other SSP staff. There have been calls to better integrate harm reduction in healthcare settings that serve PWUD [11, 36], as well as published guidelines to support collaboration [11, 37]. In our study, SSP staff put forth significant time and effort to form relationships with and educate nearby treatment programs. Additional resources are needed to help foster relationships between SSPs and treatment programs without imposing additional work for new SSPs. Future work should explore how jurisdictions can best deliver harm reduction training to improve collaboration between SSPs and the treatment community.

Integration of harm reduction interventions within SUT settings is relatively recent and has had very limited uptake: in 2017 just 7% of treatment providers in a national sample reported that their organization or practices provided sterile syringes [9]. In our study, most of the SSPs that the interviewees worked at had been operating for fewer than five years. This recency and unfamiliarity of SSP expansion in Michigan may have been a significant factor in the level of pushback we observed. Studies of expanded syringe access programs, which enable free syringe services through pharmacies, have shown that while staff were often initially reluctant to provide the intervention, participating pharmacists endorsed fewer stigmatizing attitudes towards PWUD and harm reduction over time [33, 38, 39]. Indeed, of the two national-level studies of SUT provider acceptance of SSPs, the most recent from 2017 found 78% acceptance of SSPs while the earlier 2003 study found 61% acceptance, though the samples are not directly comparable [9, 10]. There is a dearth of research on what factors contribute to the duration of pushback to harm reduction interventions. More research is needed to understand what policies, in addition to education, can hasten acceptance of evidence-based harm reduction practices.

A significant theme among SSP staff we spoke to was frustration with the lack of SUT providers, particularly MOUD providers, to which they could refer clients. SUT inaccessibility is a national crisis: in 2019 there were 21.6 million people over the age of 12 who needed SUT according to SAMSHA criteria, but just 12.2% of these received treatment [40]. Evidence-based SUT with MOUD is even more lacking: just 18.1% percent of individuals with opioid use disorder who got treatment in the prior year received MOUD [40]. Michigan is working to improve access to low-barrier SUT alongside its scale-up of harm reduction access in accordance with the 2022 national Harm Reduction Framework [41, 42], but current availability of treatment is significantly lower than demand. Less than one third of Michigan SUT providers offer MOUD [16], resulting in 35% of Michigan counties without any MOUD treatment programs for SUD at all [20]. SSP staff in our study emphasized the need for more SUT partners they felt they could trust to provide accessible and evidence-based support to SSP clients who ask about treatment. Improving the quality and quantity of SUT options in Michigan may allow for more integrated collaboration with SSPs and other harm reduction approaches.

While our study provides important insight into the relationships between SSP operators and SUT providers, our study is subject to limitations. We only interviewed SSP staff working in the state of Michigan, which has had a rapid scale-up of harm reduction services. SSP operators working in areas with a more gradual implementation process may have different experiences with treatment providers. Recall bias may have affected our data, particularly from individuals who were describing reactions to SSPs that began operating a few years prior to the study. Additionally, we did not directly assess the experiences and attitudes of providers in the treatment community. Future work to better understand the perspectives of the treatment community in this context will be beneficial. Another important perspective to consider is that of PWUD who are seeking services and navigating between harm reduction and treatment programs in Michigan. While additional perspectives are warranted, our study begins to fill the gap in understanding of harm reduction and SUT provider relationships and collaborations. Despite these limitations, our study importantly documents the intersection of harm reduction and SUT in a state with a rapid proliferation of SSPs in recent years. Our findings provide key insight into the complexities and advantages of relationship building between service providers.

## Conclusion

Our qualitative study of the experiences of SSP staff working with SUT providers during Michigan’s scale-up of harm reduction programming demonstrates the ongoing challenges gaining acceptance of evidence-based harm reduction interventions even among providers who serve overlapping communities. SSP staff described building effective collaborations with treatment providers by addressing misconceptions about harm reduction and stigma of drug use, and focusing on the complementary roles of both treatment and harm reduction in supporting people who use drugs. Collaboration between and integration of harm reduction and SUT is necessary to advance the health of people who use drugs.

## Data Availability

No datasets were generated or analysed during the current study.
